# A Diverse Tetrapod Fauna at the Base of 'Romer's Gap'

**DOI:** 10.1371/journal.pone.0125446

**Published:** 2015-04-27

**Authors:** Jason S. Anderson, Tim Smithson, Chris F. Mansky, Taran Meyer, Jennifer Clack

**Affiliations:** 1 Department of Comparative Biology and Experimental Medicine, University of Calgary, Calgary, Canada; 2 Museum of Zoology, University of Cambridge, Cambridge, United Kingdom; 3 Blue Beach Fossil Museum, Hantsport, Canada; 4 Department of Biological Sciences, University of Calgary, Calgary, Canada; College of the Holy Cross, UNITED STATES

## Abstract

The lack of fossil tetrapod bearing deposits in the earliest Carboniferous (‘Romer’s Gap’) has provoked some recent discussions regarding the proximal cause, with three explanations being offered: environmental, taphonomic, and collection failure. One of the few, and earliest, windows into this time is the locality of Blue Beach exposed in the Tournaisian deposits at Horton Bluff lying along the Avon River near Hantsport, Nova Scotia, Canada. This locality has long been known but, because the fossils were deposited in high energy settings they are almost always disarticulated, so the fauna has not been described in detail. Recent intensive collection has revealed a diverse assemblage of material, including for the first time associated elements, which permits an evaluation of the faunal constituents at the locality. Although not diagnosable to a fine taxonomic level, sufficient apomorphies are present to identify representatives from numerous clades known from more complete specimens elsewhere. The evidence suggests a diverse fauna was present, including whatcheeriids and embolomeres. A single humerus previously had been attributed to a colosteid, but there is some uncertainty with this identification. Additional elements suggest the presence of taxa otherwise only known from the late Devonian. Depositional biases at the locality favor tetrapod fossils from larger individuals, but indirect evidence from trackways and tantalizing isolated bones evidences the presence of small taxa that remain to be discovered. The fossils from Blue Beach demonstrate that when windows into the fauna of ‘Romer’s Gap’ are found a rich diversity of tetrapods will be shown to be present, contra arguments that suggested this hiatus in the fossil record was due to extrinsic factors such as atmospheric oxygen levels. They also show that the early tetrapod fauna is not easily divisible into Devonian and Carboniferous faunas, suggesting that some tetrapods passed through the end Devonian extinction event unaffected.

## Introduction

The fin-to-limb transition has been receiving a great deal of study in recent years, driven by a number of important fossil discoveries. The detailed descriptions of the Upper Devonian taxa *Acanthostega* and *Ichthyostega* [1–10] have demonstrated that the earliest tetrapods possessed numerous surprising features, such as polydactylous zeugopods and indirect evidence for the retention of internal gills. The latter, the postbranchial lamina of the cleithrum, suggests that limbs evolved before the eventual transition to land [8, 10]. On the fish side of the transition the discovery of the elpistostegid *Tiktaalik* [11–13] and new detailed study of *Panderichthys* [14–17] demonstrate the mosaic nature of the acquisition of tetrapod characters such as a neck (fish plesiomorphically retain a direct osseous connection between the pectoral girdle and rear of the skull), pelvis, and synovial joints in the bones that may have evolved into the metapodials.

These fossils, in turn, have inspired intensive exploration into the nature of the transition using the tools of developmental biology and genetics [18–20]. Work has focused on the morphological diversity of the tetrapod fossils from a functional perspective, revealing a diversity of locomotive strategies [3, 21]. These transitional stages in land locomotion, in turn, are being examined biomechanically and biochemically using various extant models such as the early diverging actinopterygian *Polypterus* [22–26], the mudskipper *Periopthalmus* [27, 28], and various species of salamander [3, 27, 29].

Confounding our appreciation for these important questions in the evolution of tetrapods is a hiatus in the fossil record. This gap spans from the classic Upper Devonian archaic fossils to the Mississippian faunas found at Greer and East Kirkton, and it is presumably within this gap, termed ‘Romer’s Gap’ for A. S. Romer who first commented upon it [30, 31], that the fossil evidence to answer these large questions is to be found. Over the years the length of the gap has been reduced so that we now know more about the late Viséan [32–37]; nevertheless, this gap has persisted, especially in the Tournaisian and early Viséan.

Recently it was suggested that ‘Romer’s Gap’ is a real biologic phenomenon [38]. This hypothesis states that, because inferred global oxygen levels for this time, shortly after a major mass extinction [39, 40], the atmosphere was unfavorable for air-breathing, terrestrial animals, and was thus a constraint preventing the tetrapod emergence onto land. As further support for the low oxygen hypothesis, the authors observed the global paucity of terrestrial arthropod fossils, which they suggested were similarly constrained by the low atmospheric oxygen levels. Following their model, terrestrialization was achieved in two phases, before and after ‘Romer’s Gap’.

This hypothesis has been criticized in a number of ways. First, more recent study demonstrated that, although oxygen levels were slightly lower than present, they had been on a global increase since the low in the Frasnian [40–42]. Another criticism, by Clack [43], observed that oceanic oxygen is a function of atmospheric oxygen. Were the atmospheric oxygen to drop, aquatic levels would drop even further, and instead of creating a constraint on terrestriality might have served as the impetus for the emergence onto land because of the relatively richer levels to be found in the air. Furthermore, were all the early Carboniferous tetrapods restricted to an aquatic environment there should be a greater likelihood of their fossils being preserved [44].

A second line of criticism derives from the fossil record itself. Since the low oxygen hypothesis was proposed, field work in the Scottish border region of the United Kingdom has revealed several rich new sources of fossils from early within ‘Romer’s Gap’ [44, 45]. These fossils include both tetrapods and arthropods, and bespeak a reasonably diverse assemblage. In other words, ‘Romer’s Gap’ appears to be an artifact of an incomplete fossil record or uneven sampling rather than reflective of an actual biological and physical phenomenon.

**Fig 1:**
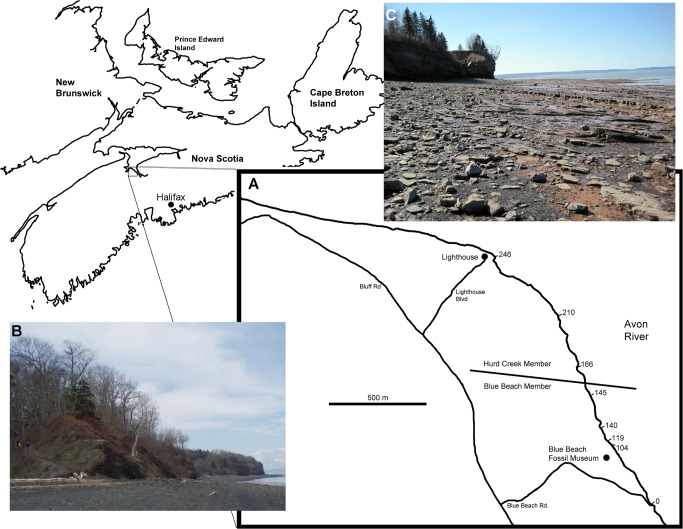
Location of Blue Beach. A, map of the shore line cliff along the Avon River Estuary where Horton Bluff Formation strata are exposed (continuing into the intertidal zone); B, Oldest beds (Hurd Creek Member) at the base of the section showing the road termination on the beach; C, the cliff and intertidal zone at Lighthouse Point (Blue Beach Member), where the majority of tetrapod specimens were recovered.

These new fossil localities fill in a great deal of ‘Romer’s Gap’ at a particular point in space and time. Here we report on tetrapod elements from the older Tournaisian vertebrate locality Blue Beach (also known as Horton Bluff), found along the shores of the Avon River at its confluence with the Bay of Fundy between Hantsport and Avonport, Nova Scotia, Canada (Fig 1). This locality has been known for a very long time [46], but for several reasons the tetrapod fossils from it have not been formally described. Fossils at Blue Beach are usually found as isolated elements, and it has taken the discovery of more complete specimens found elsewhere to place them into taxonomic context (since individual bones are diagnostic only to higher taxonomic levels). Second, the locality is difficult to work for researchers not from the area; because of the world record high tides of the Bay of Fundy the fossil bearing deposits are only accessible for a few hours twice a day. Given the cost of supporting a multiweek field trip and the isolated fragments of bone consequently produced, researchers would return to the locality infrequently. The most consistent of field parties until recently have been from the Redpath Museum (RM), building on earlier work by Don Baird [47].

However, with the regular collecting of fossil material that began in the late 1990s, which in turn led to the establishment of the Blue Beach Fossil Museum (BBFM), this greatest hurdle of prospecting for new fossils has been overcome. The BBFM is now located adjacent to the collecting site enabling the beach to be walked regularly, and the fossils revealed by each tide cycle collected and catalogued before being destroyed by the sea. This has resulted in a rapidly expanding collection of important, identifiable, fossils. Additionally, the BBFM has served as a magnet for local collectors to donate their own finds to be kept for posterity. Some highlights of the Baird, RM and BBFM collections (officially accessioned into the collections of the Nova Scotia Museum in Halifax) have been discussed in preliminary or informal reports [46–51], but none of it has been formally described in detail. We correct this longstanding deficiency here, and reveal additional specimens not previously known, in order to document the tetrapod diversity that existed in the early Tournaisian of Nova Scotia.

## Materials and Methods

Fossils from Blue Beach (Table 1) have been collected by several field parties and are housed in a number of museums in North America. The majority of the fossils discussed in the present work include those from collections in the Redpath Museum, McGill University, Montreal (RM), and the Blue Beach Fossil Museum, Hantsport, Nova Scotia (BBFM), which are accessioned into the collection of the Nova Scotia Museum in Halifax, Nova Scotia (NSM). The late Donald Baird first visited Nova Scotia in 1956 [52]. Over the next 30 years or so he made numerous collecting trips to fossil sites across the Province, including Blue Beach. None of this Blue Beach material was described in his life time. It is now housed in the Yale Peabody Museum (YPM PU), and select specimens are included in the present descriptive work. All necessary permits were obtained for the described study, which complied with all relevant regulations. This includes Province of Nova Scotia Heritage Research Permits P2002NS06 and P2004NS03 for field work conducted by JSA in 2002 and 2004, among many other collecting trips conducted over the years.

**Table 1:** List of Blue Beach specimens examined in the present study.

Specimen #	Morphology	Taxonomic referral
NSM003GF008.047	Cranial element indet.	Indet. tetrapod
NSM003GF008.054	Tibia or Fibula indet.	Indet. tetrapod
NSM003GF008.056-B	toothed element; possibly coronoid	Indet. tetrapod
NSM003GF008.053	Skull table element indet.	Indet. tetrapod
NSM005.GF045.001	Pelvis Type 1	Whatcheeriid
NSM005GF045.005	Scapulocoracoid Type 1	Embolomere
NSM005GF045.006	Scapulocoracoid Type 1	Embolomere
NSM005GF045.007	Scapulocoracoid indet.	Indet. tetrapod
NSM005GF045.009	Scapulocoracoid indet.	Indet. Tetrapod (thick)
NSM005GF045.025	?coronoid	Indet. tetrapod
NSM005GF045.034	Associated humerus Type 1, femur, distal elements	Embolomere
NSM005GF045.035A-C	Associated L. and R. Femur Type 2 and Tibia type 1	Embolomere
NSM005GF045.037	Humerus Type 1	Whatcheeriid
NSM005GF045.044	Femur indet.	Indet tetrapod
NSM005GF045.047	Femur indet.	Indet tetrapod
NSM005GF045.048A	Femur Type 4	Acanthostegid
NSM005GF045.318	Ischium?	
NSM005GF045.319	Femur indet.	“type C tetrapod”
NSM005GF045.500	Cranial elements	Indet. tetrapod
NSM005GF045.551	Dentigerous element	Indet. tetrapod
NSM005GF045.607	Vertebral centrum	Indet. tetrapod
NSM005GF045.634	Humerus Type 1	Whatcheeriid
NSM007GF004.629	Femur Type 3	Indet. tetrapod
NSM014GF036.001	Pelvis Type 2	embolomere/Eoherpetontid
NSM014GF036.005	Interclavicle	Whatcheeriid
NSM014GF036.003	Tibia Type 2	Whatcheeriid
NSM014GF036.004	Tibia Type 2	Whatcheeriid
RM 20.6703	Interclavicle	Indet. tetrapod
RM 20.6705	Jugal	Embolomere?
RM 20.6706	Scapcoracoid Type 2	Ichthyostegid
RM 20.6707	Humerus Type 2	Embolomere
RM 20.6711	Femur indet.	Indet tetrapod
RM 20.6770	Humerus Type 3	Colosteid? Rhizodont metacarpal?
YPM PU 20103	Femur Type 1	Tulerpetontid
YPM PU 20754	Humerus Type 2	Embolomere
YPM PU 23545	Humerus Type 1	Whatcheeriid
YPM PU 23550	Femur Type 1	Tulerpetontid

All photographs and interpretive drawings have been prepared specifically for this study. The fossils will be described as groups of morphotypes, focusing on the most diagnostic elements. Comparisons will be made in the Results section, and taxonomic conclusions drawn subsequent to the Discussion section. This sequencing is somewhat at odds with standard paleontological practice but is necessitated given the disarticulated and difficult to identify nature of the specimens.

Fossils from the collections of the Redpath and Blue Beach Fossil Museums were prepared mechanically using a Chicago Pneumatics or Paleotools Microjack #4 air scribe, in many cases completely three dimensionally. Note that all material recently collected including that from the BBFM is accessioned in the collections of the Nova Scotia Museum, Halifax.

### Geologic Context

All fossils were found along the western shore of the Avon River north of the town of Hantsport, Nova Scotia. The geological context was recently reviewed in detail [46], so only highlights will be presented here.

**Fig 2:**
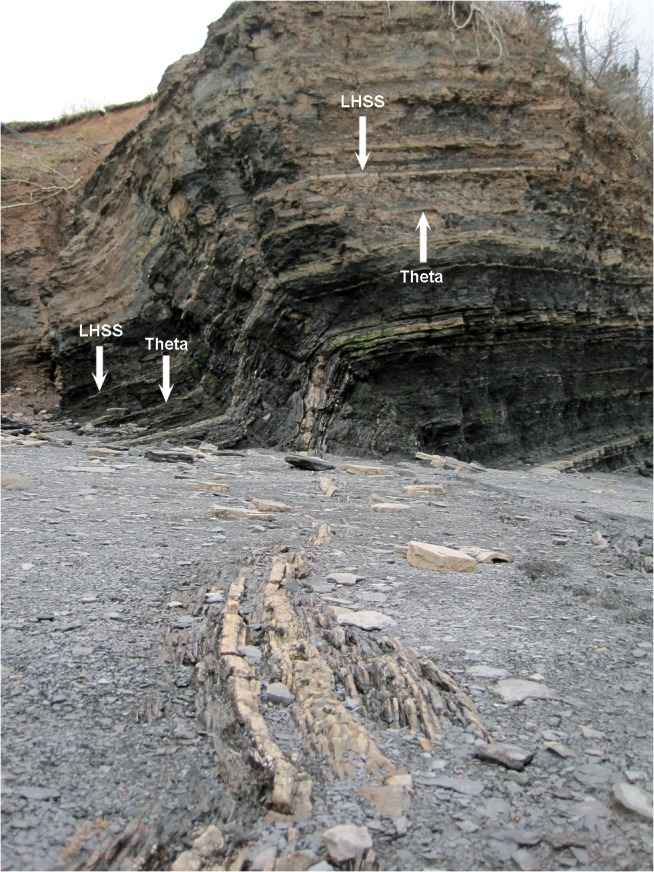
Syncline at Lighthouse Point. The resistant beds here include especially productive sandstones known informally as the ‘Theta Layer’ (**Theta**) and ‘Lighthouse Sandstone’ (**LHSS**).

The beds of the Blue Beach Member of the Horton Bluff Formation are alternating thin to moderately bedded grey silty sandstones interbedded with shales, representing transgressive and regressive cycles of near shore to intertidal facies with some occasional marine incursions. Trackways, tree stumps, and mudcracks can be found in various beds attesting to the aerial exposure of the deposits at times. The vertebrate community is dominated by fish, including basal actinopterygians, rhizodontids, acanthodids, and chondrichthyans. Gyracanths are also present but uncommon and, surprisingly given the fauna, lungfish material is rare. The fossils were initially found primarily within the sandstone units, often within lag deposits between ripples, and the overall high energy of the depositional environment has led to the disarticulation of skeletons and a bias towards larger tetrapod fossils. More recent collection has revealed the fauna to be present also in green mudstones and black shales. Because the fish are so much more common some body fossils have been found, but most are disarticulated. Many of the tetrapod fossils were collected around Lighthouse Point, the hinge of a syncline (Figs 1C and 2). Here the youngest rocks of the sequence are present, dated to early Tn3 palynomorph stage, and beds become progressively older to both the north and south, the latter with the oldest rocks in the sequence, dated to the late Tn2 stage [53]. Correlation suggests that the PC palynozone to which the Horton Bluff Formation is dated is lower than the CM zone in which most of the Scottish material has been found. However, this may have to be revised following palynological studies being carried out as part of a wider project examining the Tournaisian sequences in Britain. Several kilometers northwest of Blue Beach the Horton Bluff Formation continues down into the Upper Devonian [53] but no vertebrate macrofossils have been recovered from this area to date.

Fossils are found mainly within the intertidal river bed debris so reconstructing the specific context is difficult, but some have been located ‘in situ’; especially productive are two horizons near the lighthouse informally dubbed the ‘Theta Layer’ and ‘Lighthouse Sandstone’ (Fig 2). Because of the large amount of energy involved with the twice daily 15+ meter tides the beach changes appearance rapidly, and previously located fossils can disappear forever unless harvested immediately. This makes it extremely valuable that local collectors are able to prospect the beach frequently: many of these reported finds would never have come to light without this effort. As a result, we are reporting for the first time partially associated specimens; with time hopefully more completely articulated specimens will be located.

## Results

### Humerus Type 1

**Fig 3:**
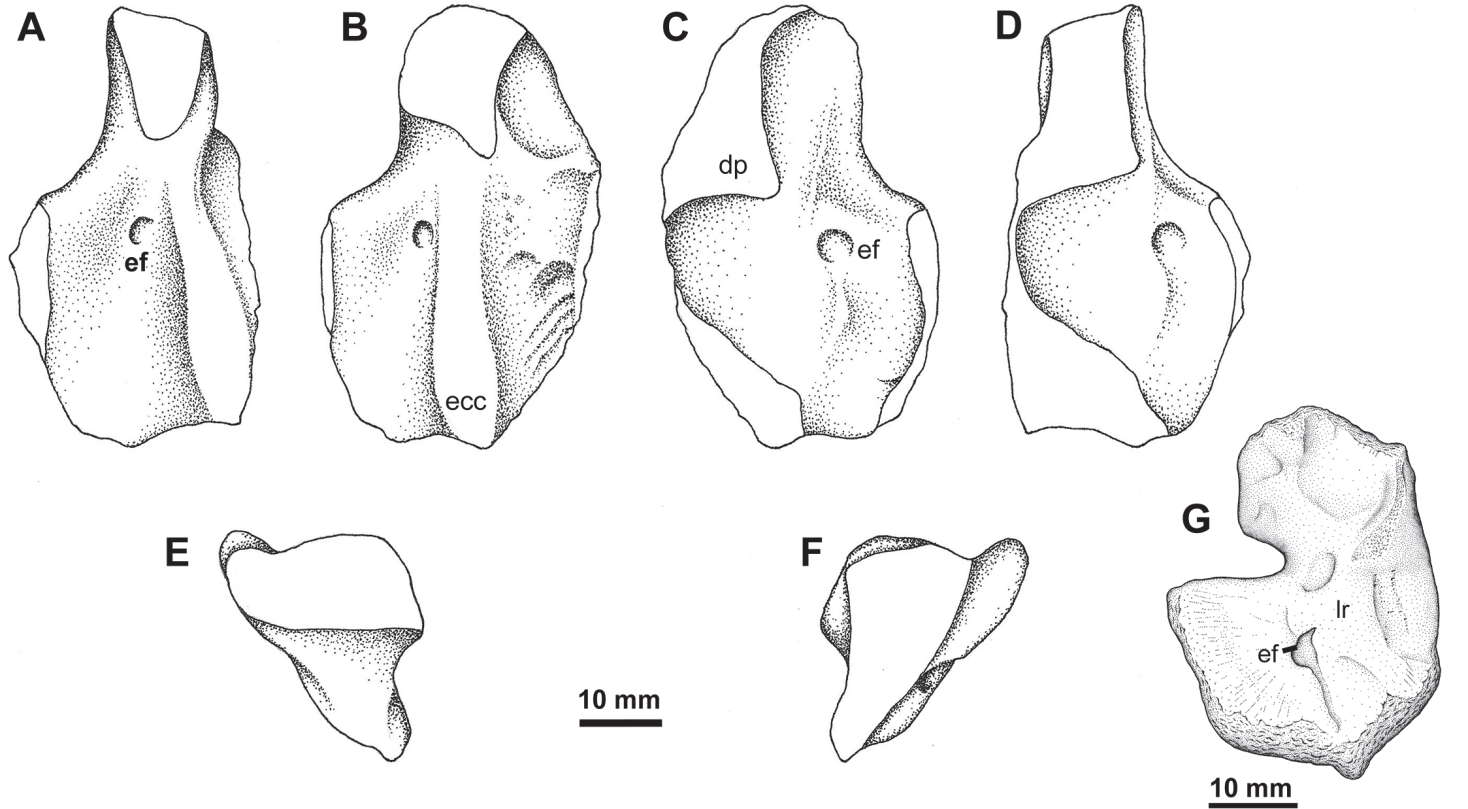
Humerus Type 1. A-F, YPM PU 23545, right humerus in A, oblique posterior, B, dorsal, C, ventral, D, oblique ventral, E, proximal, and F, distal views. G, NSM007GF004.634, small left humerus in ventral view. **Abbreviations**: **dp**, deltopectoral crest; **ecc**, ectepicondyle; **ef**, entepicondylar foramen; **lr**, longitudinal ridge.

**Fig 4:**
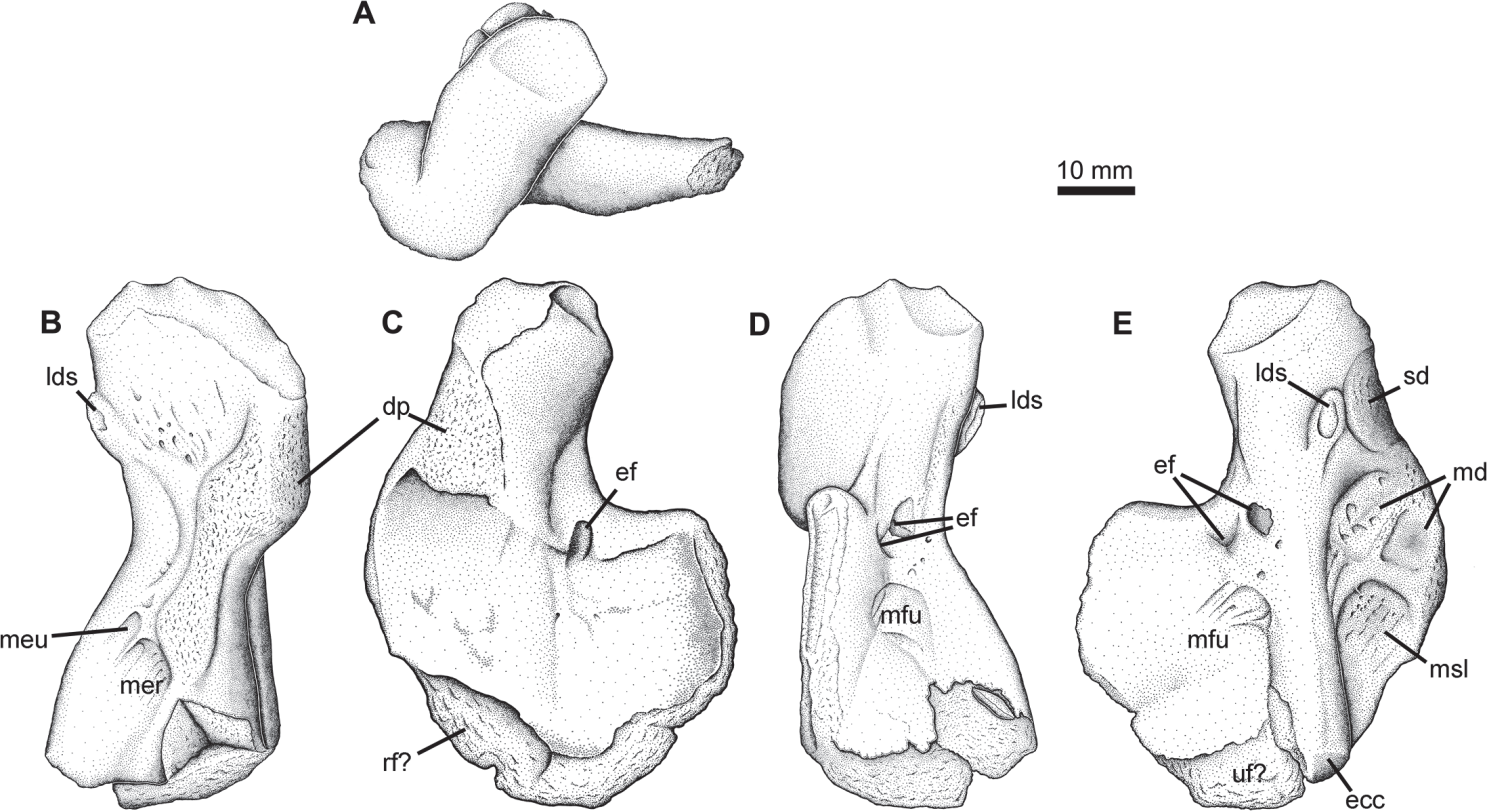
Humerus Type 1. NSM005GF045.037, right humerus in A, proximal, B, anterior, C, ventral, D, anterior, and E, dorsal views. **Abbreviations**: **dp**, deltopectoral crest; **ef**, entepicondylar foramen; **lds**, latissimus dorsi spike; **md**, m. deltoideus scar; **mfu**, m. flexor ulnaris scar; **mer**, m. extensor radialis scar; **meu**, m. extensor ulnaris scar; **msl**, m. supinator longus scar; **rf**, radial facet; sd, scapulhumeral depression; **uf**, ulnar facet.

This is a relatively recently recognized form of humerus from Blue Beach; although a specimen was collected by Don Baird in 1980 (YPM PU 23545; Fig 3A–3F) it remained unrecognized until a second was found by the BBFM, a photo of which appeared in Carroll [49] (NSM005GF045.037; Fig 4). This distinctive morphotype of humerus from Blue Beach is represented by three specimens, two right elements (one, YPM PU 23545, is 10% smaller than NSM005GF045.037) and a left that is one third the size of the others (Fig 3G), comprising a partial ontogenetic series. NSM005GF045.037 is a relatively complete specimen, but YPM PU 23545 appears to have been water worn and rolled before deposition. This is particularly evident along the anterior edge of the bone where the entire surface is unfinished and the proximal head, deltopectoral crest and distal condyles are not separated by areas of periosteal bone. The posterior edge of the entepicondyle is also worn and reduced in extent, adding to the compact appearance of the bone.

NSM005GF045.037 is a relatively massive, typically L-shaped bone, with the proximal and distal articulations offset from one another by approximately 70° (Fig 4). The proximal articulation is elongate and slightly concave posteriorly; in no specimen are the distal articular surfaces well preserved, except for a depression that presumably reflects incomplete bone ossification at the radial articulation on NSM005GF045.037, where it is visible distally on the flexor, but not extensor, surface. In flexor view, the main shaft is dominated by a large, triangular, raised scar for the insertion of the deltopectoral musculature. Distal to this the anterior edge is concave dorsally.

The entepicondyle is a broad blade that comes off the main shaft at an angle of 130°, and the proximal, thickened edge of the entepicondyle has a slight posteroproximal angulation to it. The flexor surface (Fig 4C) distal to the deltopectoral crest, including the entepicondyle, is smooth. The entepicondylar foramen is located just distal to the thickened proximal margin, just posterior of the main shaft and ectepicondyle, similar to that described in *Ossinodus* [54] and *Pederpes* [55]. On the extensor surface (Fig 4E) the foramen appears to be bifucated, with an anterior and posterior opening separated by a ridge, and on the flexor surface it exits onto a distally directed groove. This is opposite to the condition for *Ossinodus* where the foramen is bifurcated on the flexor surface, but the extensor extent of the foramen is consistent if the ridge for the Blue Beach specimen is disregarded [54]. The ectepicondyle rises dorsally from the proximal portion of the main shaft posterodistal to the latissimus dorsi spike and forms a slightly curved buttress (Fig 4E). The distal end is not completely preserved but appears to be somewhat swollen in comparison with the main shaft.

The extensor surface is, in detail, identical to that described for *Pederpes* [55] (Fig 4E). On NSM005GF045.037 a prominent, proximally-directed ‘spike’ for the insertion of the latissimus dorsi is present on the main shaft, anteriorly offset from the ectepicondylar ridge. This spike is missing from the more heavily worn YPM PM 23545. Extending anterodistally from this spike is a ridge that forms the distal margin for a concave area for the insertion of the scapulohumeralis muscle. There are two shallow depressions distal to this first ridge, defined by another ridge running from the anterior margin of the bone towards the ectepicondyle and a third running between the two anteroposteriorly directed ridges. The surface texture in this area is somewhat striated and pitted, whereas the extensor surface of the entepicondyle is smooth. Bishop [54] described this surface ornamentation as related to the insertion points for intrinsic musculature fascicles; following this author and given the location these would be related to the insertion of the deltoideus at two differentiated heads. A depression distal to the last longitudinal ridge might be associated with the insertion of the supinator longus. No supinator process is present.

### Humerus Type 2

**Fig 5:**
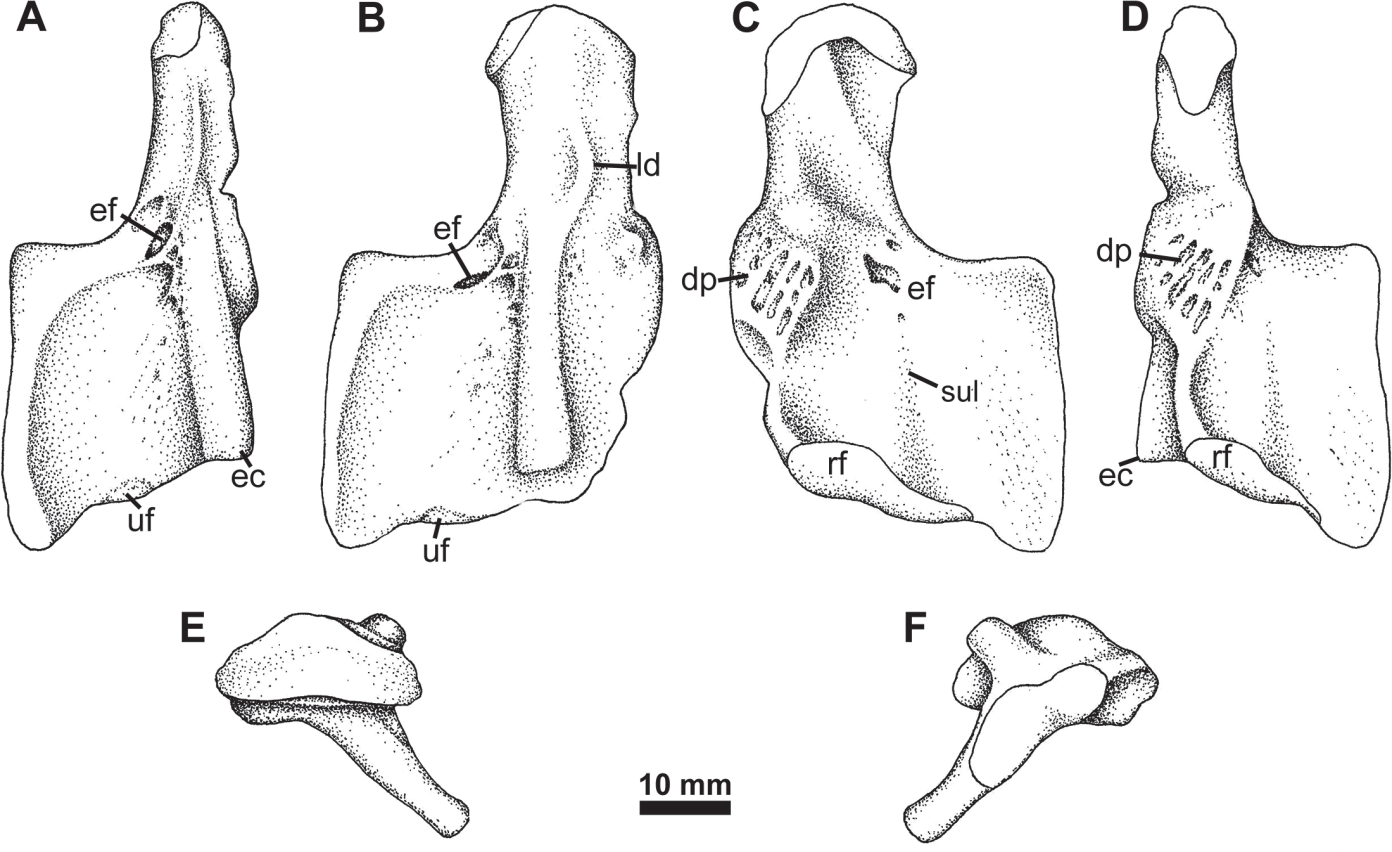
Humerus Type 2. RM 20.6707, right humerus in A, oblique posterior, B, dorsal, C, ventral, and D, oblique posterior views. **Abbreviations**: **dp**, deltopectoral crest; **ec**, ectepicondyle; **ef**, entepicondylar foramen; **ld**, latissimus dorsi scar; **rf**, radial facet; **sul**, distal sulcus; **uf**, ulnar facet.

This morphotype has been known from Blue Beach for some time, and several specimens have been found to date (Fig 5). One specimen in the collection of the Redpath Museum (RM 20.6707) was illustrated in dorsal view plane of entepicondyle and has appeared in many publications [48, 49, 51]. Compared with Humerus Type 1, Type 2 has a narrower shaft, greatly reduced deltopectoral scar, distally expanded entepicondyle, and a process for the latissimus dorsi in line with the ectepicondyle. The proximal and distal articular surfaces are offset from one another by approximately 30°. An entepicondylar foramen is located near the proximal junction between the ent- and ectepicondyles and is bounded ventrally by a fine ridge running posteriorly from the ectepicondyle on the extensor surface (Fig 5B).

**Fig 6:**
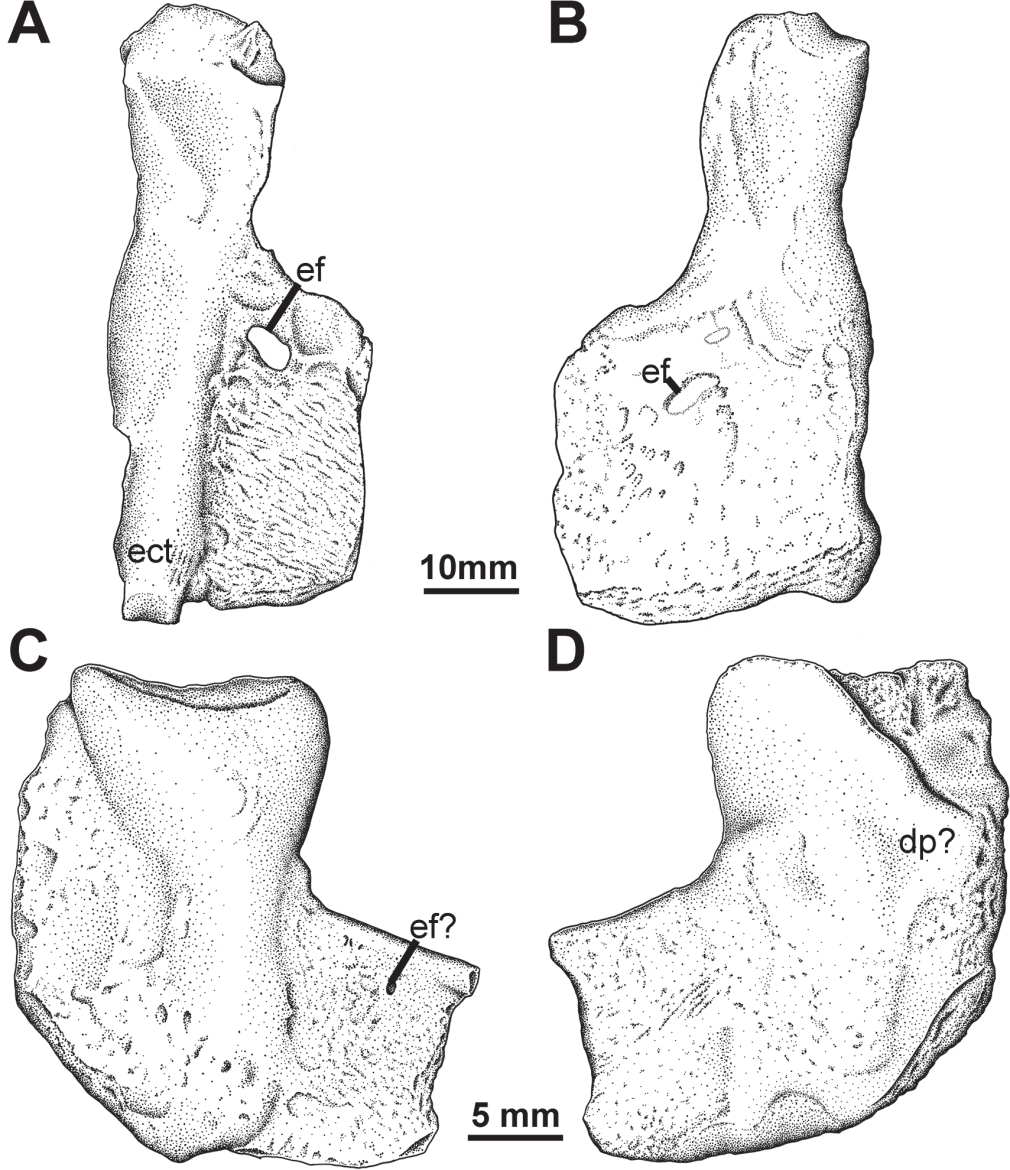
Humerus Types 2 and 3. YPM PU 20754, left humerus in A, dorsal; and B, ventral views, less areas still obscured by matrix. RM 20.6770 left? humerus in C, dorsal, and D, ventral, views. **Abbreviations**: **dp**, deltopectoral crest; **ect**, ectepicondyle; **ef**, entepicondylar foramen.

On the flexor surface (Fig 5C) two sulci can be seen running from the foramen. One is a short, shallow sulcus that extends distally from the entepicondylar foramen. The second is longer, extending much of the remaining distal length, but is directed slightly medially to run along the axis of the main humeral shaft. Both flexor and extensor surfaces are textured with a series of striations and fine pits. The proximal edge of the entepicondyle extends from the main shaft at nearly 90°. In YPM PU 20754 the ectepicondyle is a broad, rounded ridge that extends slightly anterodistally to the distal edge of the humerus (Fig 6A). It is slightly pinched-in beyond the level of the entepicondylar foramen, but for most of its length is parallel-sided. Where the posterior edge of the ectepicondyle meets the body of the entepicondyle, the surface of the bone is excavated into a number of crescentic sulci. Similar scarring is also present on the ventral surface of the entepicondyle. This has not been seen on the humeri of other taxa with a similar morphology, like the embolomeres. A supinator process is not present on any specimen.

### Humerus Type 3

The third humeral type, of which there is only a single example, is small, relatively featureless in its simple construction, compounded by weathering (Fig 6C and 6D). There is no prominent ectepicondyle, instead it appears as a swelling on the main shaft of the bone (Fig 6C). The short entepicondyle, which extends from the main shaft at a slightly oblique angle, bears its foramen in a more distal location than typical. It has a relatively prominent anterior flange that has a slightly scalloped texture at its margin. There is no torsional offset between the proximal and distal surfaces. A possible faint deltopectoral crest is present (Fig 6D), but no insertion scar for the latissimus dorsi is evident. Clack and Carroll [48] compared this type to colosteids such as *Greererpeton*, and whereas the simple construction and lack of humeral torsion is consistent, in *Greererpeton* there are multiple entepicondylar foramina located closer to the main humeral shaft, and a more distinctive ectepicondyle, so this comparison is not ideal (see Discussion) and this identification remains tentative.

### Scapulocoracoid Type 1

**Fig 7:**
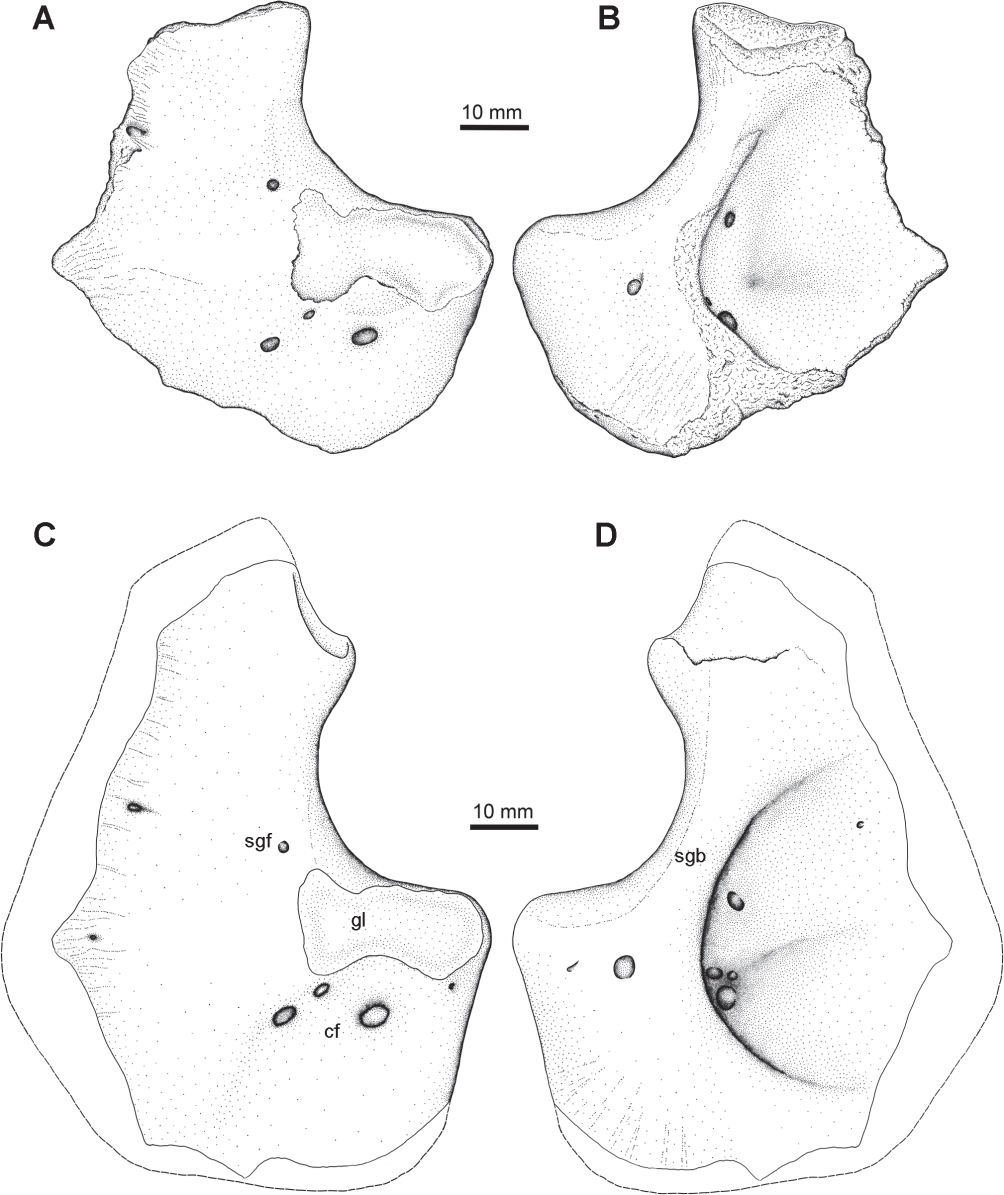
Scapulocoracoid Type 1. NSM005GF045.005, left scapulocoracoid in A, lateral, and B, medial views. Reconstruction of full extent of this morphotype from multiple specimens in C, lateral, and D, medial views. **Abbreviations**: **cf**, coracoid foramina; **gl**, glenoid; **sgb**, supraglenoid buttress; **sgf**, supraglenoid foramen.

The most common scapulocoracoid type found is rarely found complete (Fig 7); NSM005GF045.005 is the most complete specimen in the collection. It is a roughly comma-shaped single ossification, with a massive coracoid region and a robust scapular region. The lateral surface is smooth except for the anterior margin which has some short striations. There is a broad, anterior surface that is confluent with the similarly broad coracoid. The glenoid is a horizontal, anteroposteriorly elongate, and concave articulation facet, as is typical for early tetrapods, and is oriented posterolaterally. The posterior edge of the scapular region dorsal to the glenoid is thickened, contributing to the supraglenoid buttress seen on the medial surface. There is a small supraglenoid foramen, located anterodorsal to the glenoid, which appears to pierce the bone where the scapular blade is thinnest. Towards the anterior margin of the scapulocoracoid two additional, smaller, foramina can be found in positions roughly approximating foramina of *Acanthostega* identified as ‘B’ and ‘C’ by Coates [2]. Two foramina, one smaller one slightly dorsoposterior to a relatively large one, pierce the convex coracoid plate at the anteroventral margin of the glenoid, as is common for early tetrapods. A third, large, coracoid foramen is located ventral to the mid-point of the glenoid, which is constricted at this point, and at least the two larger foramina pass through the entire thickness of the scapulocoracoid.

In medial view (Fig 7B) there is a deep subscapular fossa bordered dorsally by the supraglenoid buttress and posteriorly by the thickened coracoid plate, closely similar to that described by Lebedev and Coates [56] for *Tulerpeton*. The dorsalmost part of the buttress flares anteroposteriorly and in the most complete specimen appears to form a facet for the articulation with, presumably, the cleithrum.

### Scapulocoracoid Type 2

The second type is known from a single specimen, RM 20.6706, first published by Clack and Carroll (their Fig 3B and 3D) [48]. It is an incomplete specimen, missing most of the coracoid, but it differs from Type 1 in a few important aspects. First, the posterior glenoid is continued dorsally by a thin supraglenoid process. This process was thin when first prepared, and some matrix was left in place medially to keep it in place but unfortunately, inspection of the specimen in the spring of 2013 has shown it has broken off and is now missing (JSA, pers. obs. 2013). A similar supraglenoid process has only been described in the Devonian tetrapod *Ichthyostega* [1]. Second, the supraglenoid foramen is located in the supraglenoid buttress dorsal to the glenoid towards the point above anterior third of its total length. In other respects, the specimen compares with the Type 1 scapulocoracoid in the location of foramina and general form.

### Femora

Femora are among the more commonly found tetrapod elements at Blue Beach, but they frustratingly lack diagnostic features. Nevertheless, discrete morphotypes can be identified. All of the following descriptions rely on the nomenclature of Lebedev and Coates [56] and Coates [2].

#### Femur Type 1

**Fig 8:**
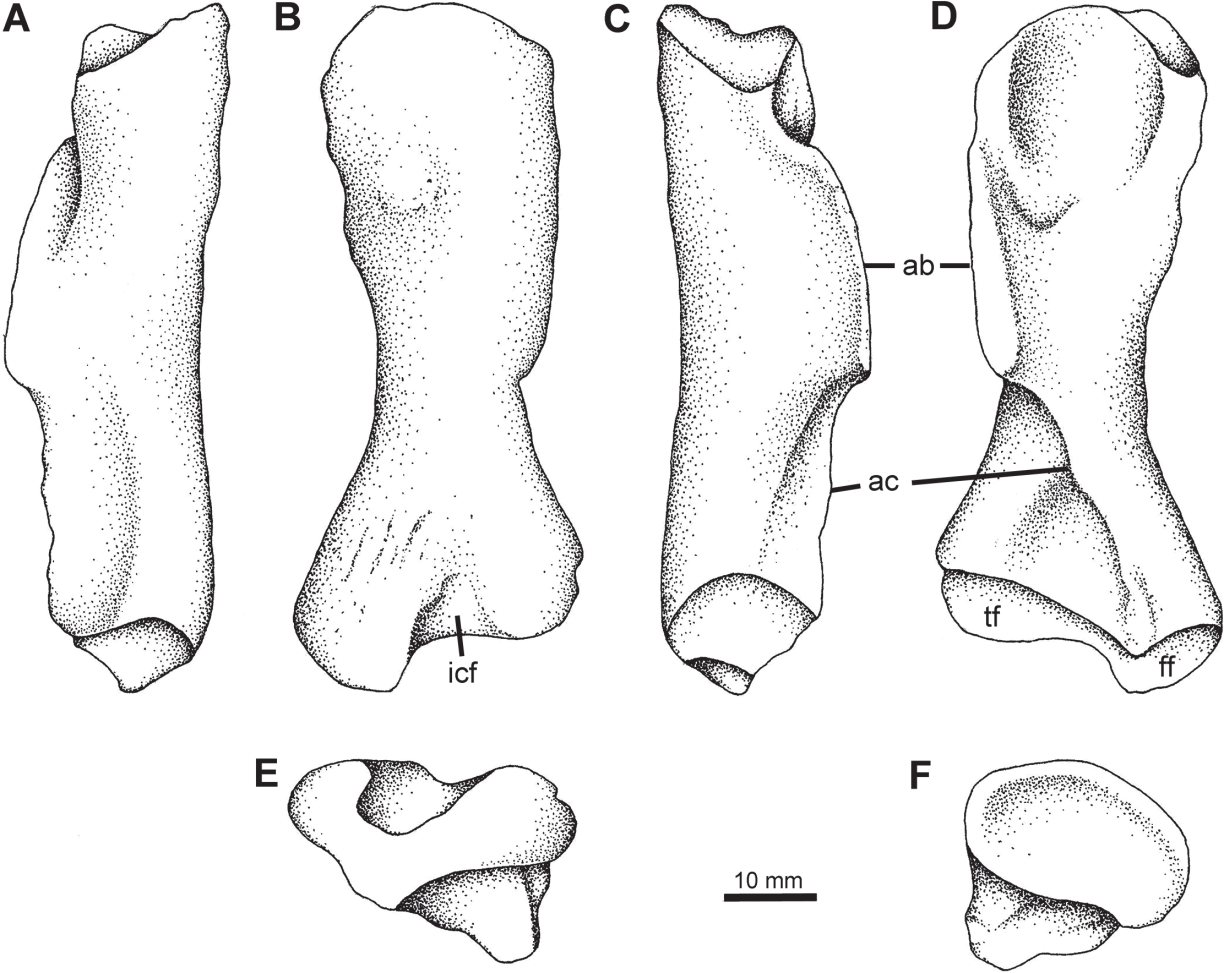
Femur Type 1. YPM PU 23550, right femur in A, posterior, B, dorsal, C, anterior, and D, ventral, E, proximal, and F, distal views. Scale bar equals 10 mm. **Abbreviations**: **ab**, adductor blade; **ac**, adductor crest; **ff**, fibular facet; **icf**, intercondylar fossa; **tf**, tibial facet.

**Fig 9:**
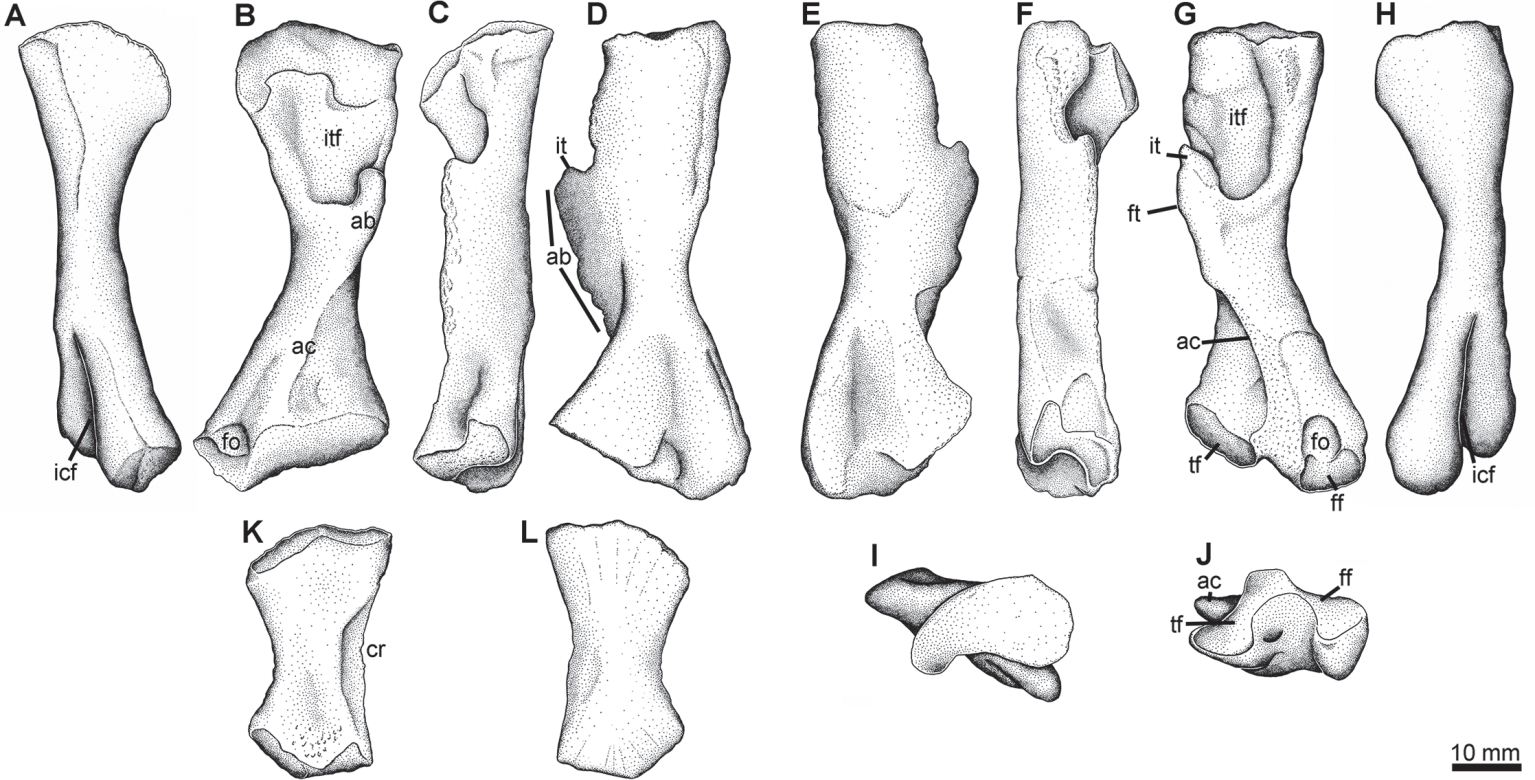
Femur Type 2 and tibia Type 1. NSM004GF045.034A-C, right and left femora (Type 2) and left tibia (Type 1) found in association. A-D, I, J, left femur in A, posterior, B, ventral, C, anterior, D, dorsal I, proximal, and J, distal views. E-H right femur in E, dorsal, F, anterior, G, ventral, and H, posterior views. Left tibia in K, flexor (ventral), and L, extensor (dorsal) views. **Abbreviations**: **ab**, adductor blade; **ac**, adductor crest; **cr**, thin anterior crest; **ff**, fibular facet; **fo**, fibular fossa; **ft**, fourth trochanter; **icf**, intercondylar fossa; **it**, internal trochanter; **itf**, intertrochanteric fossa; **tf**, tibial facet.

The first morphotype is by far the most commonly seen, and several examples have been illustrated in preliminary or informal reports. Don Baird collected several examples of this morphotype. The first of these, YPM PU 20103, was collected in 1968. A cast was made of this specimen, and this was figured but not described by Clack and Carroll (their Fig 5F) [48]. A larger and more complete specimen collected by Baird in 1971, YPM PU 23550 (Fig 8), shows that the cast specimen has been significantly crushed. This has flattened the proximal and distal ends, displaced the adductor blade anteriorly and reduced the depth of the intertrochanteric fossa. As a result the femur appears to be much broader than it would have been in life. In contrast, YPM PU 23550 is a much more cylindrical bone, with a deep adductor blade and a pronounced tuberosity on the dorsal surface, and has a number of features that make it distinct. YPM PU 23550 has a sigmoidal shape to its diaphysis, where the long axis bows towards the adductor blade, opposite to the bow seen in femur Type 2 (Fig 9), and proximally there is an inflection to bring the proximal head back in that direction (so convex to concave moving proximally from the midshaft). The shaft is relatively robust, and the adductor crest passes transversely to approximately the fibular facet, whereupon it passes more distally longitudinally.

The proximal articulating surface is roughly oval in outline and orientated proximally. Its unfinished surface is excavated into a deep trough that extends across the end of the bone. In ventral view the rim of the articulating surface is convex, with the posterior edge more distal than the anterior. The adductor fossa is relatively small and forms a shallow depression marked by pits and striations on the proximal ventral surface of the femur. These pits are deepest along the distal margin of the fossa. The adductor blade forms the anterior wall of the adductor fossa. The blade is clearly separated from the proximal articulating surface by finished bone. The internal and fourth trochanters are indistinct but presumably they formed the proximal end of the adductor blade as in *Acanthostega* [2] and *Tulerpeton* [56]. The rectangular blade is a thickened tongue of finished bone that projects ventrally along the long axis of the femur, for about one third of its length. In anterior view, the blade is angled down slightly so that the distal end lies below the proximal end. It terminates abruptly with the ventral and distal edges almost at right angles to each other forming a distinct step, rather than taper, between the adductor blade and the adductor crest. The crest is a sharp low ridge that extends diagonally across the distal half of the ventral surface of the femur to the edge of the distal articulating surface. Here it forms the intercondylar ridge between the fibular and tibial condyles.

The distal articulating surface is roughly v-shaped in outline. It is angled proximoventrally so that much of it is visible in ventral view. The dorsal surface is marked by a deep intercondylar fossa that lies directly above the intercondylar ridge. The ridge and fossa divide the articulating surface into two unequal halves: the anterior, tibial condyle is approximately twice the size of the posterior, fibular condyle. The fibular condyle is the more distal of the two and it forms a distinct extension of the posterodistal end of the femur that is clearly seen in dorsal view. The fibular fossa is shallow. On the proximal end of the dorsal surface there is a large bulbous swelling behind the proximal articulation and lying above the proximal end of the adductor blade. A much smaller swelling on the *Tulerpeton* femur, termed the central prominence [56], is thought to mark the insertion of the puboischiofemoralis internus muscle.

#### Femur Type 2

An associated left-right pair with associated tibia (NSM005GF045.035a-c) found in and prepared out of the same block, presumably belonging to the same individual (Fig 9), is a very close match with *Tulerpeton* in a number of aspects. The proximal articulating surface is anteroposteriorly long and curved around the extensive intertrochanteric fossa. The internal trochanter is present on the adductor blade as a proximally directed process, with a distinct rounded notch setting it off from the femoral shaft. The fourth trochanter is located at the same point on the adductor blade on the flexor surface. The robust, relatively triangular adductor blade extends approximately one third of the length of the femur, and tapers into a thin adductor crest for most of the remaining extent of the femur, onto the buttress in the popliteal area that forms the posterior margin of the tibial facet. Unlike *Tulerpeton*, the transition between adductor blade and crest in the Blue Beach specimen is more gradual, lacking the distinct stepped appearance in *Tulerpeton*, although this could be due to weathering seen in the Devonian form. NSM005GF045.035 has a relatively gracile shaft, which is convex towards the adductor blade, making the shaft more bowed than sigmoidal.

Distally the tibial condyle is located slightly more proximally than the fibular condyle. Both condyles are offset from one another by a deep intercondylar fossa. Both condyles are crescentic in shape, and the tibial is slightly saddle-shaped from its continuation onto the popliteal buttress. A deep fibular fossa can be found on the flexor surface, and a similar fossa, bounded medially by the adductor crest, can be found proximal to the tibial condyle.

#### Femur Type 3

**Fig 10:**
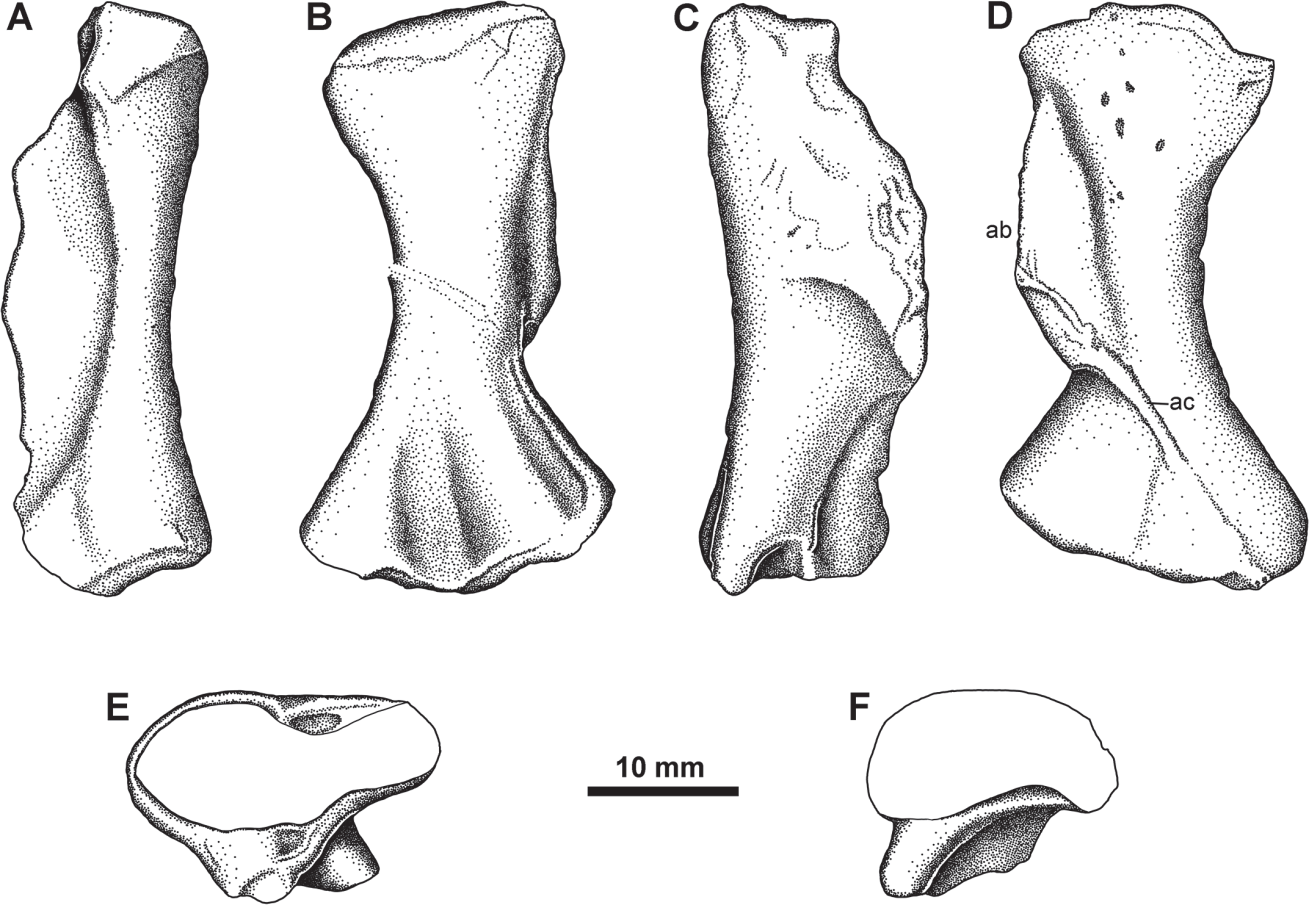
Femur Type 3. NSM007GF004.629, left femur (reversed for comparison) in A, posterior, B, dorsal, C, anterior, and D, ventral views. **Abbreviations**: **ab**, adductor blade; **ac**, adductor crest.

NSM007.GF004.629 is an oddity in the collection (Fig 10), and comes from the older deposits near the entrance to Blue Beach (Blue Beach Member) approximately 33m up section Fig 1B), rather than the cove below the lighthouse (Hurd Creek Member; Fig 1A). It looks squat, but this could be the result of rolling wear. The adductor blade is worn but appears to occupy most of the length of the bone. The adductor crest passes transversely, even after it reaches the fibular facet, rather than passing distally at the fibular facet as in the other morphotpes. The diaphysis is straight to slightly sigmoidal distally. The intertrochanteric fossa is relatively shallow.

#### Femur Type 4

**Fig 11:**
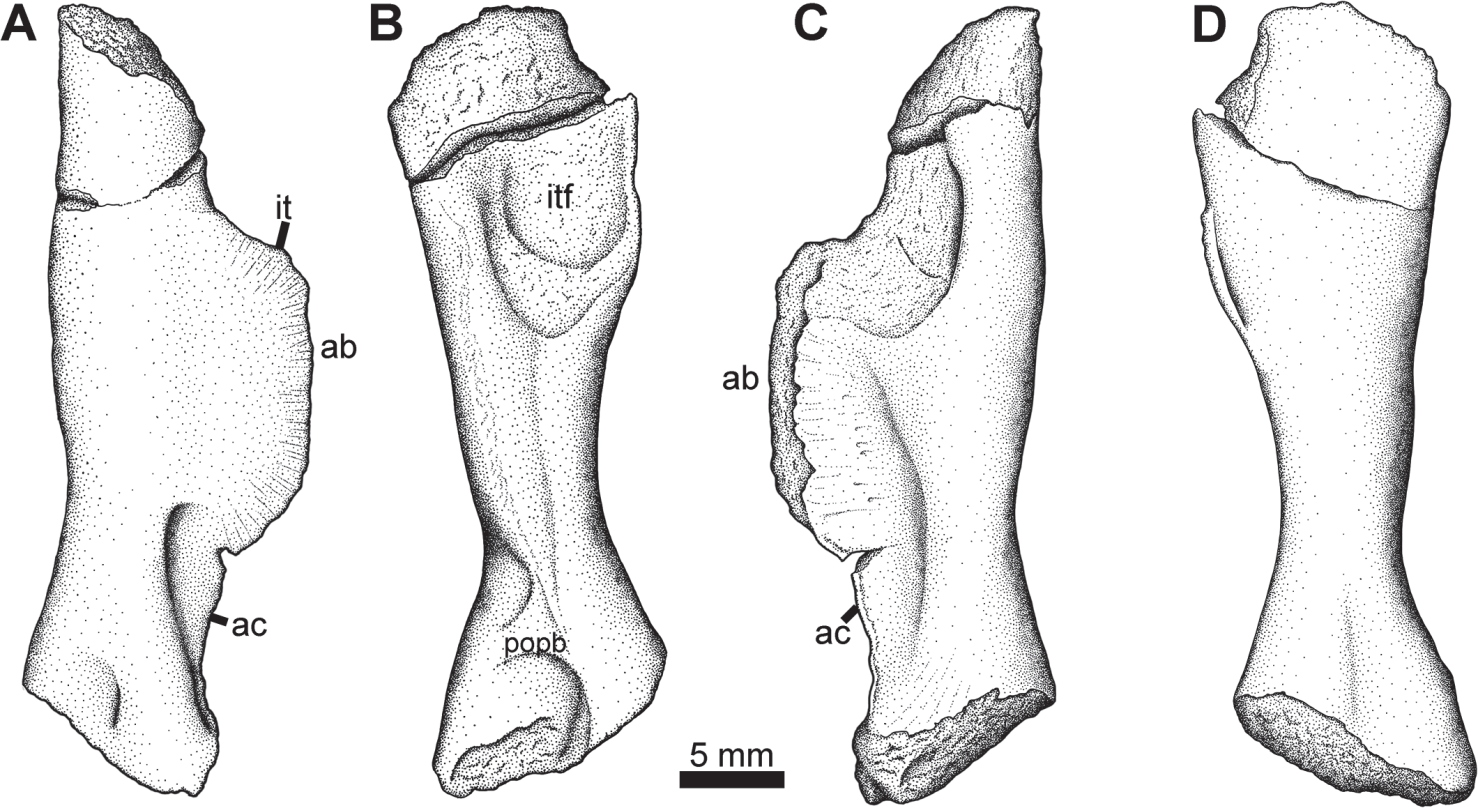
Femur Type 4. NSM005GF045.048-A, right femur in A, anterior, B, ventral, C, posterior, and D, dorsal views. **Abbreviations**: **ab**, adductor blade; **ac**, adductor crest; **it**, internal trochanter; **itf**, intertrochanteric fossa; **popb**, popliteal butress.

A final femur type, represented by a single specimen (NSM005GF045.048A; Fig 11), is much smaller than the others (Table 2). The proximal and distal condyles are somewhat eroded or not fully ossified. It is extremely similar in morphology to *Acanthostega* [2]. The relatively enormous adductor blade is more elongate than seen in the others, spanning nearly half the total length of the element, and passes nearly straight distally rather than having a transverse component. Distally it ends abruptly, stepping down onto a lower adductor crest that continues down onto the popliteal buttress. There is a deep popliteal fossa on the flexor surface, and a prominent intertrochanteric fossa proximally. Proximally on the adductor blade is the internal trochanter, creating a slight notch on the proximal margin of the blade. This femur differs from *Acanthostega* in the shape of the curve of the adductor blade. In *Acanthostega*, the blade curves such that its greatest height away from the femoral shaft is located distal to the midpoint of the blade; in NSM005GF045.048A the adductor blade is more even along is length, descending towards the femoral shaft at either extremity.

**Table 2:** Principle measurements (in mm) of select femora from Blue Beach.

Specimen #	Morphology	Length	Width (proximal)	Width (midshaft)	Width (distal)
YPM PU 23550	Femur Type 1	76	28	15	32
NSM005GF045.035A	Femur Type 2	68	24.4	11.5	30.25
NSM005GF045.035B	Femur Type 2	62.45	18.7	12.8	25.9
NSM005GF045.048A	Femur Type 4	37.65	12	6	11.35

### Tibia Type 1

A left tibia, found associated with two Type 2 femora (NSM005GF045.035c; Fig 9K and 9L), is less than half the length of the femur and is somewhat dorsoventrally flattened. This is taken to be its natural condition considering the undistorted nature of the femur, and lack of obvious signs of compression on the surface of the bone. The proximal and distal surfaces are expanded, with the proximal the wider. There are two pronounced fossae on the flexor surface defined by several ridges. A thincrest is present on the anterior surface. The proximal articulating facet is curved to match the tibial condyle of the femur. The distal facet matches the description for the tibia of *Tulerpeton* [56], in being somewhat bent in the shape of an ‘L’ by the confluence of a buttress with the articulating surface.

### Tibia Type 2

**Fig 12:**
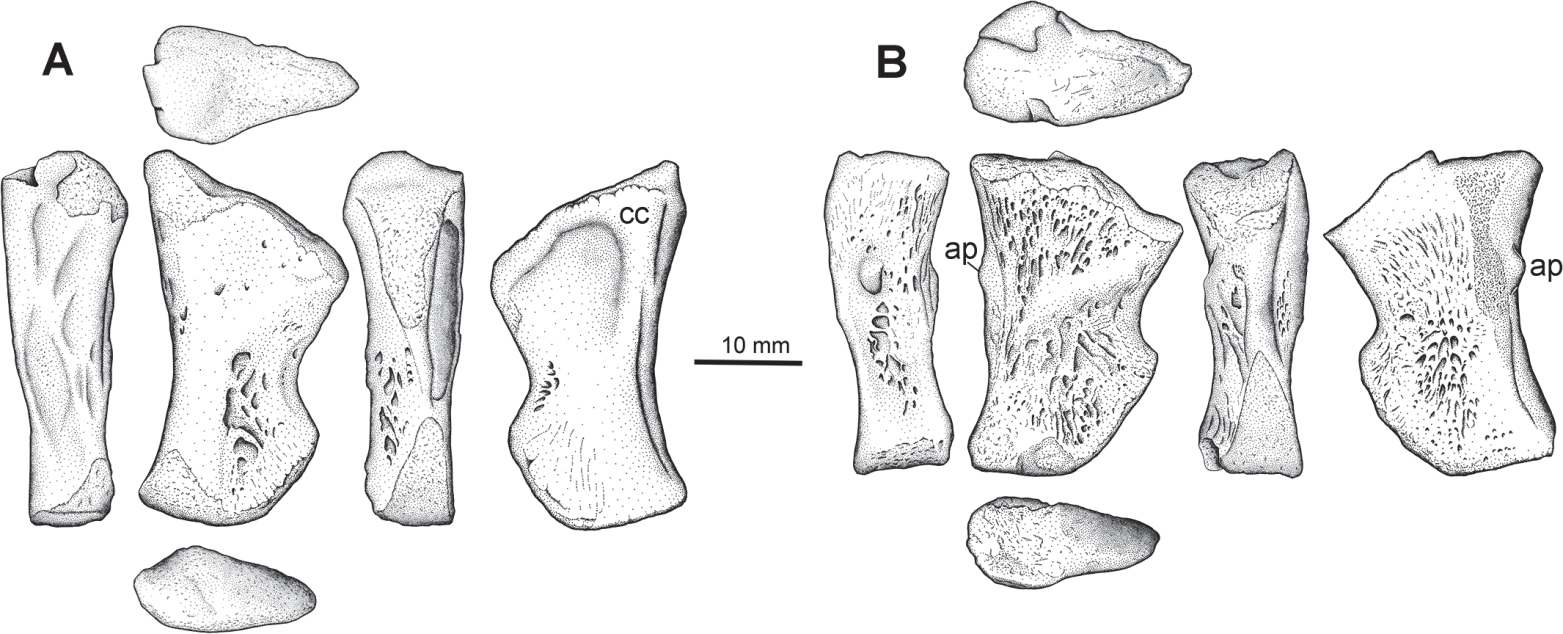
Tibia Type 2. NSM014GF036.003 (A) and NSM.014.GF.036.004 (B). Right tibiae each in anterior, flexor, posterior, and extensor views. **Abbreviations**: **ap**, anterior process; **cc**, cnemial crest.

Two additional tibiae in our study collection (NSM014GF036.003 and NSM014GF036.004) are consistent with a *Pederpes*-like whatcheeriid, being very similar in shape and detail to both *Pederpes* and *Ossinodus* (Fig 12). These tibiae differ strongly from the one described with femur Type 2. They are more flattened, and have a deeper notching along the posterior margin, which rotates the distal condyle into a more proximal location. The anterior margin lacks the thin crest; instead, each tibia is thickened along this margin, defined in part by a thickened ridge along the extensor surface, and is concave. Within the concavity can be found a series of striations and one knob like projection just proximal to the midpoint of the shaft. The extensor surface is lightly striated in ornament and bears a robust cnemial crest but the flexor surface has deep ridges running proximodistally, interrupted by a prominent ridge that passes from proximoposterior to distoanterior along the proximal half of the surface. The two Type 2 tibiae appear to differ from each other somewhat, but these differences appear to be attributable to the greater degree of weathering seen in Fig 12A, expressed both in terms of rounding of edges and wearing off of the surface ornament details, and allometry. However, future morphometric study may reveal they differ in degrees of robustness; for now we take a conservative approach and group them. An associated fibula found with one of these tibiae is similar to that of *Pederpes* in being equal in length to the tibia.

### Pelvis Type 1

**Fig 13:**
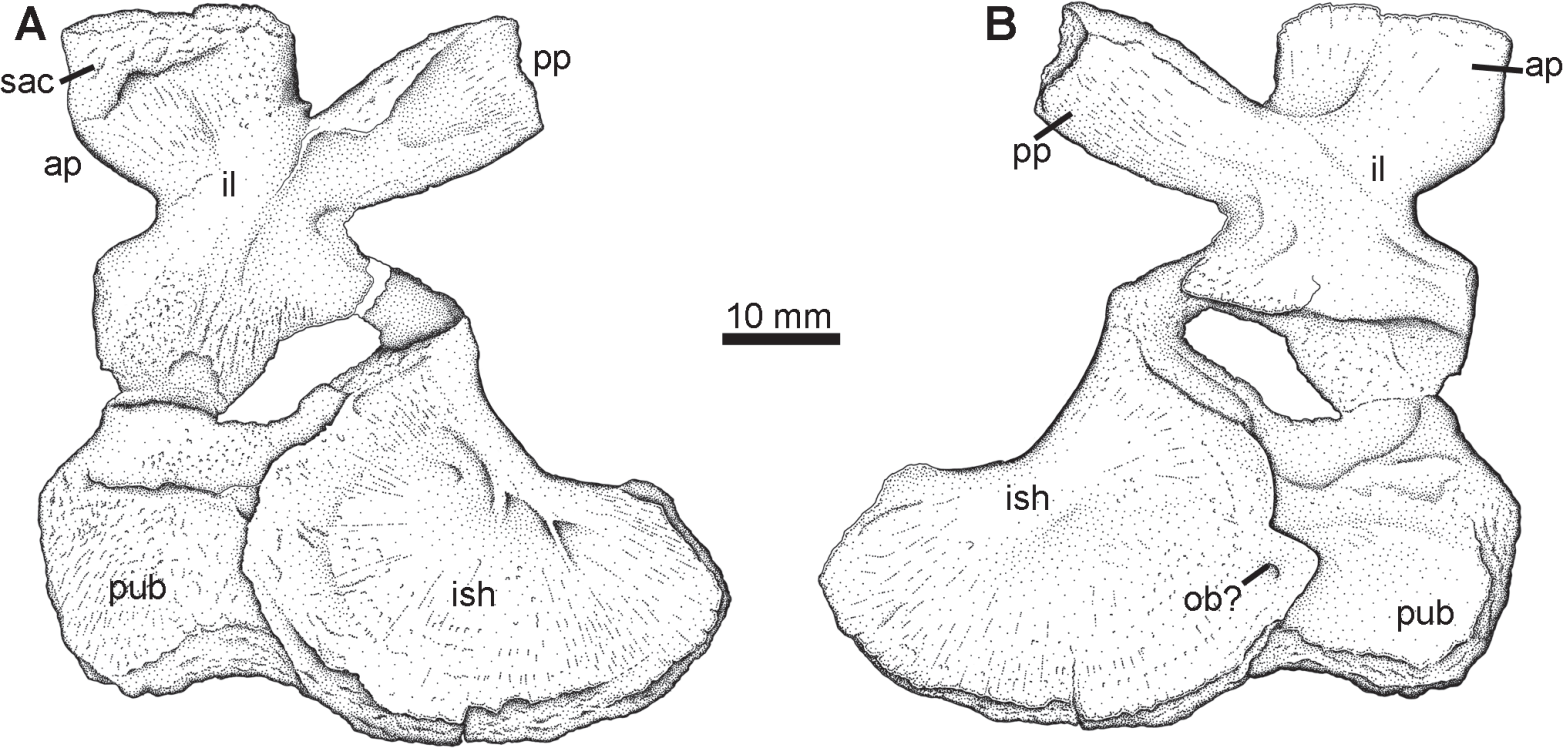
Pelvis Type 1. NSM005GF045.001, right pelvis in A, medial, and B, lateral views. **Abbreviations**: **ap**, anterior process; **il**, ilium; **ish**, ischium; **ob**, obturator foramen; **pp**, posterior process; **pub**, pubis; **sac**, sacral rib facet.

The first of two diagnostic pelvic morphotypes is represented by a complete right girdle half (NSM005GF045.001; Fig 13). The ilium has the double processes common in early tetrapods, but with a morphology distinctive of whatcheeriids. The dorsal process, for articulation with the sacral rib, is fan shaped with a narrower neck, as described for *Pederpes* [55]. The posterior iliac process is also narrower at its base and broadens distally. Like *Pederpes* but unlike *Whatcheeria* [57], these two processes are separated by a notch rather than overlapping. The posterior process is also somewhat concave medially but this could be due to compression.

The pubis and ischium are both ossified and tightly sutured together, unlike *Pederpes* which apparently lacks an ossified pubis. The broadly crescentic ischium has a thickened dorsal margin, which bears a thin ridge on the medial surface, and it is dorsally curved, whereas in *Pederpes* this margin is straight. The smaller pubis is roughly rectangular, but all margins are somewhat worn except for the tight suture with the ischium. The acetabulum is not well preserved except on the ventral surface of the ilium, which has a robust dorsal buttress. No obvious obturator foramen is present. A potential small candidate lies on the pubis along its suture with the ischium but it does not seem to perforate fully through the bone. The surface of the ilium is smooth, whereas the ischium and pubis bear light radiating striations.

### Pelvis Type 2

**Fig 14:**
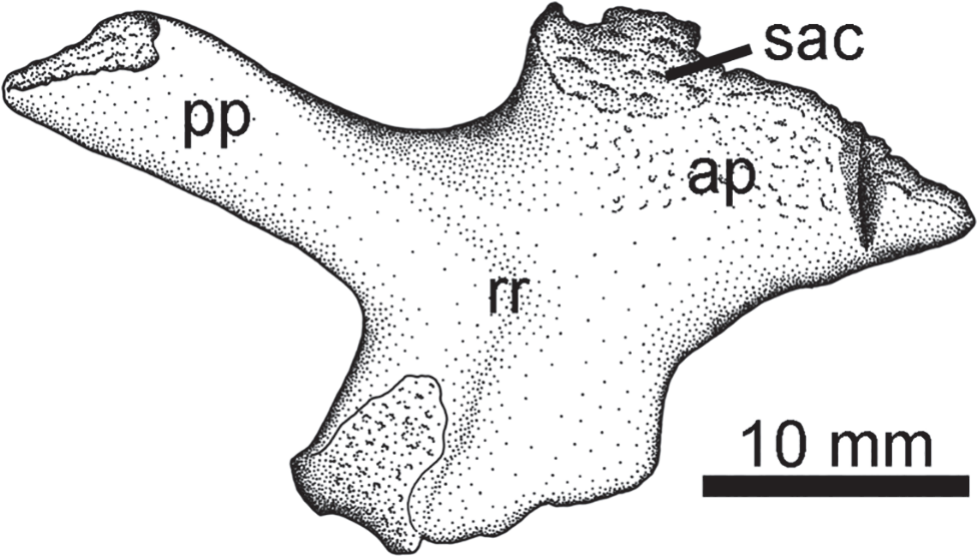
Pelvis Type 2. NSM014GF036.001, left ilium in medial view. **Abbreviations**: **ap**, anterior process; **pp**, posterior process; **rr**, robust ridge; **sac**, sacral rib facet.

The second diagnostic pelvic element is an ilium (NSM014GF036.001) preserved in medial view found during fieldwork in 2004 (Fig 14). This ilium also bears two processes, one dorsal towards the sacrum and one posteriorly directed. However, the posterior process is parallel sided and lacks the distinctive distal flaring seen in the first type. Furthermore, a robust ridge extends from the posterior acetabular region dorsally, then curves posteriorly to continue down the length of the posterior process. This is similar to what is seen in *Eoherpeton* from the Carboniferous of Scotland [58].

## Discussion

Despite the fragmentary nature of the fossils from Blue Beach, sufficient material is present to make an estimation of taxonomic diversity present at the locality. These elements are currently diagnostic to relatively high taxonomic levels and because of a paucity of articulated or even associated material (see below), it cannot be said whether two given elements belong to the same or different species. We will therefore take the conservative approach and assume all elements related to the same higher taxon belong to the same species, pending the discovery of additional fossil material.

### Whatcheeriid

A whatcheeriid is clearly present in the Horton Bluff Formation fauna. Humerus Type 1 is nearly identical to that described for *Pederpes*. It also preserves muscle scars similar to those recently described in *Ossinodus* [54], but differs from the latter in having an apparent bifurcation in the insertion of the deltoideus. The overall form of the humerus, in terms of placement of the entepicondylar foramen, morphology of the deltopectoral crest, and shape of the entepicondyle is common among Carboniferous stem tetrapods including *Baphetes*, *Pederpes*, and *Crassigyrinus*. However, in *Baphetes* and *Crassigyrinus* the entepicondyle projects from the main humeral shaft at an oblique angle (more so in *Crassigyrinus* than *Baphetes*). There is also a spike for the latissimus dorsi in *Baphetes* but as previously observed [55] it is placed differently in that taxon from that in *Pederpes*. Type 1 also shares with *Crassigyrinus* and *Pederpes* a unique condition in which the entire anterior edge is unfinished. In the humeri of other taxa, including *Acanthostega* [2], the anterior edge is covered with periosteal bone between the proximal articulation and the deltopectoral crest and the deltopectoral crest and the distal articulation for the radius. In *Crassigyrinus*, *Pederpes*, and humerus Type 1, this periosteal covering is absent. This absence is not size related as the very much smaller humeri probably attributable to *Doragnathus* [45] have a periosteal covering along most of the anterior edge. It is notable that the humeri of *Pederpes* [55] and *Whatcheeria* [57] are very different from one another, despite the similarities of their skulls and aspects of their postcranial skeletons. The differences suggest rather different modes of locomotion in the two genera.

Pelvis Type 1 is clearly consistent with a *Pederpes*-like whatcheeriid. *Whatcheeria* and *Pederpes* both possess a posterior process that is waisted proximally and flares distally [55, 57]. In *Ossinodus* [59, 60] the waisting is much less apparent, however. Unlike *Whatcheeria*, but like *Pederpes*, the specimen lacks marginal fimbriation. The Blue Beach whatcheeriid may differ from *Pederpes* in retaining an ossified pubis.

Tibia Type 2 is also supportive of the presence of a *Pederpes*-like whatcheriid. They differ strongly from tibia Type 1, as outlined above.

**Fig 15:**
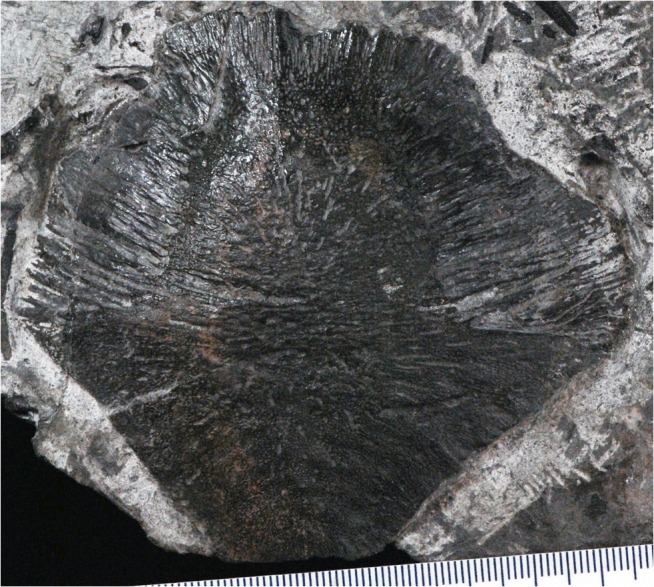
Interclavicle. NSM.014.GF.036.005, undescribed interclavicle found in situ in the Theta Layer sandstone below the lighthouse in 2002. The morphology is consistent with a whatcheeriid and with similar elements from Dora, Scotland.

Similarly suggestive of a *Pederpes*-like whatcheeriid but not diagnostic is an interclavicle found in situ within a resistant sandstone layer (Theta Layer) a few centimeters thick directly below the lighthouse in 2002 (Fig 15). The dermal ornamentation is indistinguishable from that of *Pederpes*, and similar to a recently described interclavicle from Dora [45]. A detailed consideration of the range of variation of interclavicle morphology present at Blue Beach is the subject of additional study now underway.

### Embolomere/ ‘Reptiliomorph’

**Fig 16:**
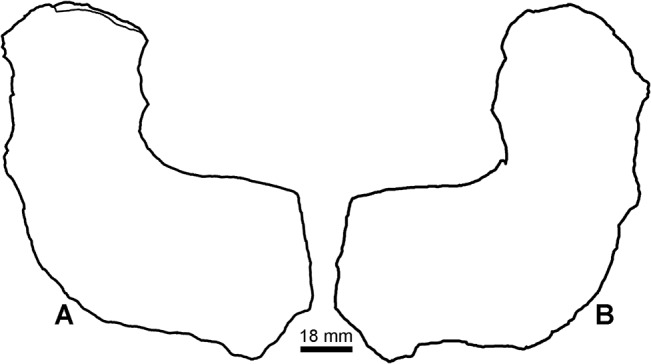
*Eoherpeton* humerus. New outline drawings of humerus NMS 1993.56.21 (formerly NUZ 78.1.58) in the plane of the entepicondyle. A, ventral, and B, dorsal views.

Evidence is equally strong for the presence of a second tetrapod in the Blue Beach fauna based on comparison of the limb bone morphology. Humerus Type 2 has previously been compared with *Tulerpeton* and *Eoherpeton* [48]. Information presented here permits broader comparison. *Tulerpeton* shares the downturned posterodisal margin of the entepicontyle with the Blue Beach form, as does the later occurring *Proterogyrinus* [61]. In contrast, the proximal margin leaves the main shaft at an oblique, rather than a nearly 45°, angle. The entepicondylar foramen is also placed more laterodistally in *Tulerpeton*, whereas the foramen is in the same location in *Eoherpeton*. As depicted previously *Eoherpeton* also has an oblique angle between the entepicondyle and humeral shaft. This is apparent however, due to the orientation of the element in the photograph from which the outline drawing was made (Fig 16). Additionally, the humerus of *Eoherpeton* is much larger than any found so far at Blue Beach so, whereas Blue Beach Type 2 appears closely affiliated, it is not the same species.

Femur Type 1 shares with that of *Tulerpeton* a shallow adductor fossa, a straight adductor blade orientated along the long axis, a deep intercondylar fossa and, most notably, a ‘central prominence’ [56] on the dorsal surface immediately behind the proximal condyle. It differs from the *Tulerpeton* femur in being more robust, with broader proximal and distal condyles and a broader shaft.

**Fig 17:**
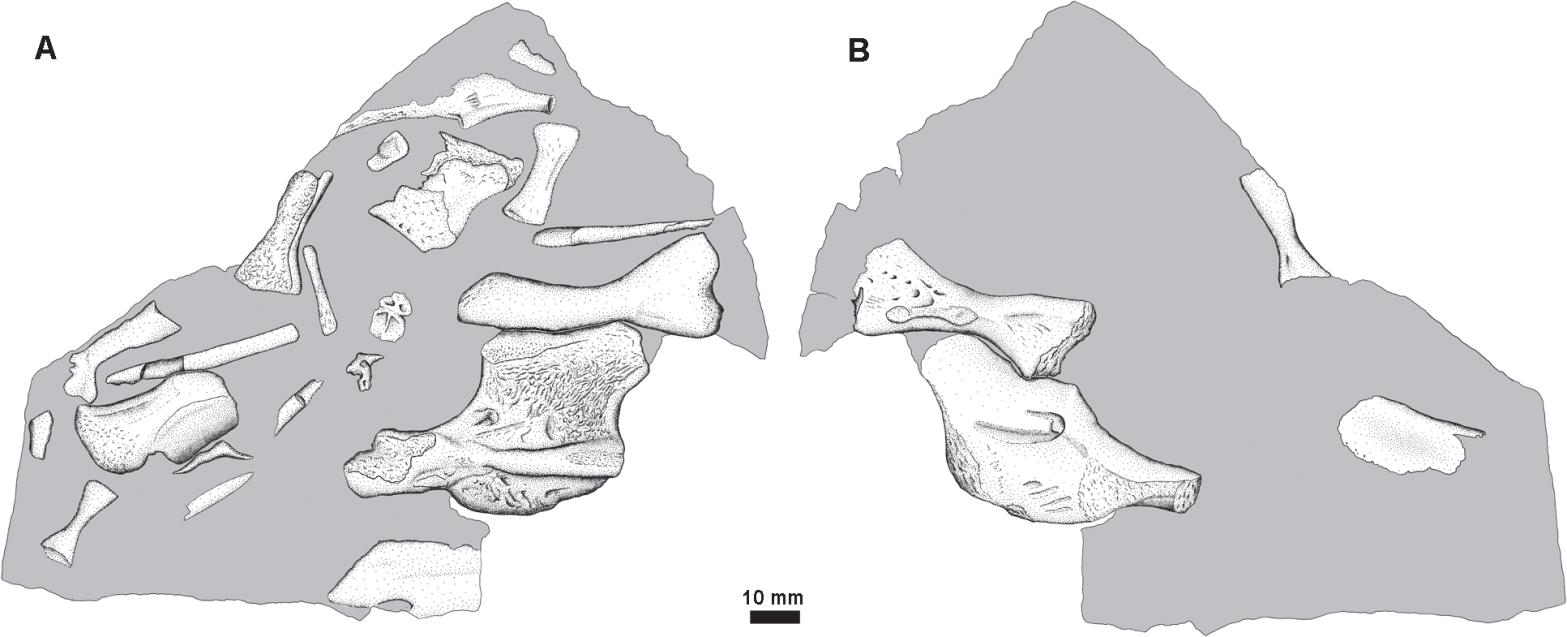
Associated material from Blue Beach. NSM005GF045.034, the ‘Sherm Block’ (for collector Sherman Williams) of associated front and hind limb material.

Femur Type 2 and the tibia Type 1 found associated with the paired right and left examples of this type (NSM005GF045.035A-C) are also consistent with an animal similar to *Tulerpeton* and *Eoherpeton*. Further support for this identification comes from another of the few specimens from the locality with tetrapod elements in close association, which places a humerus Type 2 (identified as embolomere above) next to a femur Type 2 (Fig 17; NSM005GF045.034). Of course, the elements are disarticulated, and the depositional environment fairly high energy so these associations might be happenstance; however, neither element shows signs of having been transported far. It is hypothesized here that the elements became disarticulated in place [46]. The Type 2 femur shares with *Tulerpeton* a distinct notch between the adductor fossa and adductor blade. This is most clearly seen in anterior and posterior views. However, it differs from *Tulerpeton* in having an obliquely orientated adductor blade and it lacks the ‘central prominence’.

Scapulocoracoid type 1 is consistent with a tetrapod similar to *Tulerpeton* and *Eoherpeton*, but the closest match is with the later occurring Carboniferous embolomere *Proterogyrinus* [61]. This similarity is seen in the overall extent of ossification of the element, the distribution and number of foramina, and shape of the glenoid. Additionally, the glenoid has the same posterolateral placement, but in *Proterogyrinus* there appears to be a slight posteroventral orientation to the long axis of the glenoid, which is more horizontal in the Blue Beach specimen (as far as can be determined from the incomplete material). In this feature the Blue Beach specimen is more consistent with *Greererpeton*; however, *Greererpeton* lacks the massively ossified coracoid plate seen in both *Proterogyrinus* and the Blue Beach morphotype [62]. Along with the details of humeral morphology the details of the scapulocoracoid may suggest that the Blue Beach embolomere/‛reptilimorph’ shares closer affinity with the later occurring embolomeres than more contemporary specimens.

### Devonian forms

*Tulerpeton* is a Devonian tetrapod that retains a polydactylous autopod [56]. Nevertheless, it shares a number of features consistent with embolomeres that occur later in the Carboniferous. Evidence is also present at Blue Beach for a form with closer relationship to an *Acanthostega*-*Ichthyostega*-grade tetrapod. Scapulocoracoid Type 2 is most closely matched with *Ichthyostega* in possessing the supraglenoid process and with *Acanthostega* in much of the patterning of the foramina. Femur Type 4 is an extremely close match with *Acanthostega*, but does differ in the curvature of the adductor blade.

### Colosteid?

Humerus Type 3 was compared with *Greererpeton* [48], although this identification is not perfect, as discussed above. An even closer match for this humerus is with a specimen recently discovered from the Tournaisian-aged deposits in the Scottish borderlands region at a locality known as ‘Willie’s Hole’ [44] by the late Stan Wood. This as yet undescribed specimen, nicknamed ‘Ribbo,’ in the collection of the National Museum of Scotland in Edinburgh forms part of a nearly complete, articulated skeleton, which will potentially provide a very accurate taxonomic identification. Unfortunately, it is at present unknown whether it is a colosteid, another known taxon, or a new taxon.

The humerus pertaining to ‘Ribbo’ still maintains distinctive morphological landmarks lacking in humerus Type 3, including a humeral ridge, scar for the insertion of the latissimus dorsi, and a distinct deltopectoral crest (although a rugosity is present in the expected area). This absence in the Type 3 humerus could be due to the obvious high degree of weathering. The distal displacement of the relatively small ‘entepicondylar foramen’ is particularly concerning. An alternative identification for this element may be a pectoral fin element of a rhizodont, other elements of which are plentiful at Blue Beach [63]. A match may be seen in the ulna of *Strepsodus* [64]. Consistent with this identification is the presence of a concave proximal head and generally cynlindrical shape of the element. Contrary to this is the presence of a postaxial process (identified as the entepicondyle above); rhizodonts lack such an element [64]. Instead, the distal surface of this element in rhizodonts is flared into two unequal articular surfaces for the radials (lacking in humerus Type 3) which can also be seen in the species described from Ducabrook [65]. The ulna of the Devonian *Sauripterus* is an approximate match for overall shape, with a rounded shaft lacking distinct processes seen in tetrapod humeri, scalloping along the anterior margin, and a posterior projection that superficially resembles an entepicondyle such as that seen in Fig 6C and 6D [66]. However, there is more of a proximal expansion in this latter feature in *Sauripterus* than is seen in the distal flange in humerus Type 3 as well as the twin distal columnar articulations with the radials. The morphology of the ulna in *Letognathus* is currently undescribed in the literature, but what is known from the collection is unlike that of *Sauripterus* or humerus Type 3 (CFM pers. ob.), so assignment to a distal rhizodont fin element is not fully satisfactory. It is hoped that with the description of ‘Ribbo’ and further study of the Blue Beach rhizodonts clarity can be brought to this element’s identification. Until then, the presence of a colosteid is not definitively established. The absence of a colosteid at Blue Beach would eliminate an approximately 20 million year gap in the fossil record until the appearance of the ‘colosteid-like’ tetrapod from the St. Louis Formation [67], and the slightly younger definitive colosteids *Greererpeton* at Goreville [68] and *Deltaherpeton* at Delta [69] in the Viséan.

### Implications for ‘Romer’s Gap’

Like the localities of the Scottish-English borderlands, Blue Beach provides strong evidence against ‘Romer’s Gap’ being the result of a physical phenomenon causing a hiatus in the fossil record. Instead, the rich tetrapod fauna present at Blue Beach supports the evidence from the UK suggesting that the paucity of specimens from this time period reflects either depositional processes or collecting failure [44]. It now seems that, whenever we discover rare windows into this time period, we find numerous fossil tetrapods reflecting a rich diversity of forms.

Ultimately, this dispute might hinge on a semantic argument over the definition of ‘terrestrial’. We know however that, notwithstanding the primitive construction of most of the fossil tetrapods present at Blue Beach, some of these animals was at least occasionally terrestrial from the footprint data from various beds at the locality [46]. These data additionally point to fossil tetrapods not currently known from body fossils, including potentially small, clawed forms and other trackways similar to ichnotaxa attributed to temnospondyls elsewhere [70]. At present, the Blue Beach fauna is clearly composed of forms firmly on the tetrapod stem, consistent with other localities of similar age.

Excitingly, the Blue Beach fauna appears to demonstrate that, as far as can be determined, some tetrapod taxa may have been largely unaffected by the Late Devonian extinction event. This window into the earliest Carboniferous belies a dichotomy between ‘Devonian tetrapods’ and ‘Carboniferous tetrapods’; both ‘faunas’ are present at Blue Beach. Specimens consistent with ichthyostegids, acanthostegids, and *Tulerpeton* represent the Devonian forms, whereas all remaining specimens are present at other early Carboniferous localities in Scotland, at Greer (West Virginia), and Delta (Iowa). Whatcheeriids in particular appear to be common in early Carboniferous deposits globally. It was recently reported [71] that elements similar to whatcheeriids were present in the Late Devonian deposits of Red Hill, PA; if correctly attributed this not only suggests that the tetrapod fauna was less affected than previously supposed by the Late Devonian extinction event, but strongly refutes the suggestion that ‘Romer’s Gap’ was a biotic event caused by low oxygen levels or other causes. It also implies that there are no clear boundaries between Devonian and Carboniferous faunas. It remains to be seen whether this is due to the still coarse level of sampling; it may be that the extinction impact and recovery was sufficiently rapid not to have been sampled as yet.

A final, equally tantalizing, implication of these fossils is the question of the timing of the origin of amniotes. If *Tulerpeton* represents the earliest occurrence of embolomeres (as appears to be suggested by the close similarity between humeral and femoral morphology), and if embolomeres represent stem-group amniotes as suggested by some studies [56, 72], then the amniote-amphibian divergence may predate the Carboniferous, as suggested by some molecular clock estimates [73–75], although some of those could be overestimates [76]. More work on these fossils is clearly required to examine this possibility in more detail.
